# Meta-Analysis Approach identifies Candidate Genes and associated Molecular Networks for Type-2 Diabetes Mellitus

**DOI:** 10.1186/1471-2164-9-310

**Published:** 2008-06-30

**Authors:** Axel Rasche, Hadi Al-Hasani, Ralf Herwig

**Affiliations:** 1 Max-Planck-Institute for Molecular Genetics, Department of Vertebrate Genomics, Ihnestrasse 63-73, D-14195 Berlin, Germany; 2 German Institute for Human Nutrition, Potsdam-Rehbrücke, Arthur-Scheunert-Allee 114-116, D-14558 Nuthetal, Germany

## Abstract

**Background:**

Multiple functional genomics data for complex human diseases have been published and made available by researchers worldwide. The main goal of these studies is the detailed analysis of a particular aspect of the disease. Complementary, meta-analysis approaches try to extract supersets of disease genes and interaction networks by integrating and combining these individual studies using statistical approaches.

**Results:**

Here we report on a meta-analysis approach that integrates data of heterogeneous origin in the domain of type-2 diabetes mellitus (T2DM). Different data sources such as DNA microarrays and, complementing, qualitative data covering several human and mouse tissues are integrated and analyzed with a Bootstrap scoring approach in order to extract disease relevance of the genes. The purpose of the meta-analysis is two-fold: on the one hand it identifies a group of genes with overall disease relevance indicating common, tissue-independent processes related to the disease; on the other hand it identifies genes showing specific alterations with respect to a single study. Using a random sampling approach we computed a core set of 213 T2DM genes across multiple tissues in human and mouse, including well-known genes such as *Pdk4*, *Adipoq*, *Scd*, *Pik3r1*, *Socs2 *that monitor important hallmarks of T2DM, for example the strong relationship between obesity and insulin resistance, as well as a large fraction (128) of yet barely characterized novel candidate genes. Furthermore, we explored functional information and identified cellular networks associated with this core set of genes such as pathway information, protein-protein interactions and gene regulatory networks. Additionally, we set up a web interface in order to allow users to screen T2DM relevance for any – yet non-associated – gene.

**Conclusion:**

In our paper we have identified a core set of 213 T2DM candidate genes by a meta-analysis of existing data sources. We have explored the relation of these genes to disease relevant information and – using enrichment analysis – we have identified biological networks on different layers of cellular information such as signaling and metabolic pathways, gene regulatory networks and protein-protein interactions. The web interface is accessible via .

## Background

Type-2 diabetes mellitus (T2DM) is a rapidly increasing disease with more than 170 million afflicted persons worldwide (constituting more than 90% of all diabetic patients). T2DM poses a huge burden for the health care systems and is, thus, subject to intensive biomedical research. T2DM is a multigenic disease involving a high number of susceptibility genes and causes alteration of an entire network of genes. Several environmental and nutritional risk factors have been identified for T2DM the most relevant being obesity where multiple molecular mechanisms have been proposed to link obesity to insulin resistance and beta cell failure [1]. Increased availability of food and reduced physical activity as a consequence of modern lifestyle are the main drivers for an anticipated epidemic increase in T2DM patients in the next years.

In the pathopysiology of T2DM, impaired insulin sensitivity and glucose intolerance are early phenomena, leading to hyperglycemia, hyperlipidemia and, eventually, to a failure of pancreatic beta cells to produce and secrete a sufficient amount of insulin. However, most genes and their associated molecular network contributing to the onset and course of the disease are yet unknown.

Genetic variation in the context of diabetes has already been extensively studied, leading to numerous candidate genes. Studies on transgenic and knock-out mice have been valuable to dissect the regulatory network of genes implicated in insulin action and body weight control [2,3], however, monogenic variants contribute only to a minority of T2DM cases. In contrast, the polygenic nature of T2DM is now well established and several polygenic mouse models including NZO, BTBR etc. have been studied to analyze diabetes susceptibility on a more complex genetic background [4]. Linkage analyses have shown that several quantitative trait loci interact with each other and with the environment to elicit obesity syndromes that are potentially diabetic. Several recent genome-wide association studies have identified novel candidate genes for T2DM but the effect of these variants on disease susceptibility is generally low, with odds ratios mostly around 1.5 [5-11].

Multiple studies on the transcriptome level have been performed that emphasize the diversity of the disease and the complex pathophysiological interactions between different tissues, including fat, muscle, liver, pancreatic beta cells and brain [1]. In several human studies, tissue biopsies from diabetic and normoglycaemic individuals have been profiled [12,13]. In mouse studies differences in diet or mouse strains have been used to identify distinct expression profiles [14-16]. Complementary ChIP-on-Chip studies reveal the associated gene regulatory network of important transcription factors (TFs) active in the relevant tissues [17,18]. In the context of the onset of diabetes, several studies on the proteomic level have revealed differential expression of intracellular proteins as well as of secretory proteins in adipose tissue [19]. Despite the availability of these large amounts of data, their common content as well as their specific differences, in particular in gene sets between human and rodent studies, has not yet been systematically evaluated.

The goal of this meta-analysis approach is to generate additional value by combining the above-mentioned individual studies and by extracting consistent information. Several meta-analyses studies have been previously applied within other disease domains, such as cancer [20] or Alzheimer [21] using different types of data. With respect to T2DM some recent approaches have been published: In Tiffin et al. several computational prediction methods have been combined in order to identify a common set of T2DM genes [22]. The authors assessed the accordance of the prediction methods resulting in a candidate gene list of 99 different genes. For type-1 diabetes mellitus a web-resource has been set up that tracks the expression behavior of genes in several tissues [22]. Liu et al. have applied enrichment analysis to previously defined gene sets and protein-protein interactions using data from different species and tissues from the Diabetes Genome Anatomy Project [23] and identified a subnet of insulin signaling proteins and nuclear receptors [24]. In contrast to Rhodes et al., Sun and Liu et al. our approach is not limited to transcriptome studies [7,24,25]. We have accumulated data from different levels of molecular interaction such as genetic information using knock-out mice and single nucleotide polymorphisms (SNPs), gene regulatory and gene expression information as well as information on protein signaling and protein interactions. In order to reduce technical bias of transcriptome measurements we restricted this data type to experiments that were performed on the Affymetrix GeneChip platform. Similar to Liu et al., we combined the relevant tissues such as liver, muscle, adipose tissue and pancreas [24] since T2DM has physiological consequences in several parts of the body. Furthermore, a more global view on T2DM has been achieved by involving mouse as well as human data because the available mouse models address specific aspects of the disease and it is unclear, whether these mice have diabetes for the same reason as humans do.

Using a Bootstrap [26] scoring approach we computed a core set of 213 genes that show significant disease relevance in the data sets under study. Here, we used the gene expression profiles along with qualitative data comprising literature, genetic and proteomic sources. Besides known genes our approach exhibits a large fraction (128) of yet barely characterized novel candidate genes. These genes have been further validated in the functional context of networks and exhibit high potential for understanding pathways and pathway crosstalk associated with T2DM. By applying gene set enrichment analyses we inferred the deranged parts of the physiology using gene ontology terms [27], common pathway resources [28-30] and information on the associated gene regulatory network [17,18,31].

The meta-analysis approach is generic and can be used as a template for studies in other disease domains. It has been completely implemented in the software platform R using the BioConductor package collection [32-34]. Our T2DM-GeneMiner web resource [35] allows the user to access the information that was gathered and to assess diabetic potential for any human or mouse gene of interest.

## Results

We present the identification of the T2DM candidate genes, the comparison to previously published T2DM gene lists and results from association and linkage studies as well as the identification of associated molecular networks on different levels.

### Annotation, preprocessing and categorization of data

We used Ensembl (version 39) as the annotation reference database. Homology between human and mouse genes was derived via BioMart. The total number of genes under study comprises 15,277 Ensembl mouse genes representing the union of the homologue genes from all data sources. An overview about the T2DM specific datasets is given in Table 1.

**Table 1:** Overview on the datasets used for the T2DM meta-analysis approach.

**Data set**	**Category**	**Species**	**Tissue**	**Study research question**	**Reference**
StumvollGoldstein2005	qualitative	human		medical review about T2DM	[1]
DeanMcEntyre2004	qualitative	human		medical review about selected candidate genes	[48]
OMIM	qualitative	human		medical review about T2DM	[44]
PubMedGeneRIF	qualitative	human/mouse		text mining in the NCBI geneRIF	[49]
KOmiceJAX	qualitative	mouse		mouse models with phenotype T2DM	[2]
NandiAccili2004	qualitative	mouse		mouse models with phenotype "Insulin Resistance"	[3]
ChenHess2005	qualitative	rat	fat	secretory proteins in adipose tissue	[19]
MoothaGroop2003	quantitative	human	muscle	patients with T2DM/impaired glucose tolerance and controls	[13]
GuntonKahn2005	quantitative	human	pancreas	patients with T2DM versus controls	[12]
LanAttie2003	quantitative	mouse	fat/muscle/liver/pancreas	diabetic mice versus controls	[15]
BiddingerKahn2005	quantitative	mouse	fat/muscle/liver	diabetic mice versus controls	[14]
NadlerAttie2000	quantitative	mouse	fat	diabetic mice with different level of hyperglycaemia	[16]

Several of the available resources are based on microarrays. Each individual microarray study was normalized using the GC RMA method of the R/BioConductor package [33,34,36]. Recently, it has been reported that the remapping of the oligoprobes, as an alternative to the annotation given by the chip provider, enhances data analysis to a significant extent [37]. Using this mapping we indeed observe differences in annotation resulting from recent changes in genome annotation. Annotation comparisons showed improvement of reproducibility and specificity (data not shown). Re-annotation affects the main fraction of genes, for example in the remapped Mouse Genome U74A version 2 platform the top ten genes of our candidate list are represented by oligoprobe sets of 8 to 15 probes (11 with annotation of the chip provider) with an average of 13 probes (*Serpina1a *11 probes, *Ybx1 *8, *Pdk4 *16, *Cstb *14, *Adipoq *15, *Agt *14, *Lgals1 *11, *Serpine2 *15, *Mt2 *10, *Gpi1 *16 probes).

### Identification of T2DM candidate genes – generality versus specificity

Numerical scores were computed for all genes in each individual study, the scores were summarized and the summarized scores were compared against a random sample at the 99.9 percentile as described in Materials & Methods. This procedure determines a cut-off score value of 3.05 and identifies a set of 213 genes with a score exceeding this cut-off.

**Figure 1:**
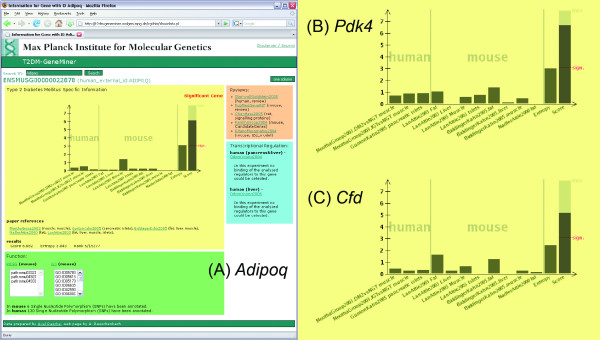
Screen-shot of the web tool showing results on different genes with different amount of available information: (A) *Adipoq*, (B) *Pdk4 *and (C) *Cfd*.

Randomly, we would expect 15 out of the 15,277 genes to exceed the threshold. Cutting at the 99 percentile results in 943 genes (expecting 153 by chance), cutting at the 98, 97, 96 and 95 percentiles would result in 1352, 1587, 1792 and 1972 selected genes (305, 458, 611 and 764 randomly expected genes). Thus, the ratio of detected vs. expected significant genes increases with percentile of the random sample from 2.6 to 14.2, indicating a necessary precondition for the validity of our selection procedure [see Figure 1 in Additional file 1]. Since we have analyzed data from multiple tissues in human and mouse, it is likely that for some cases an individual experiment is dominating the score, for example, if the gene is active only in a single tissue. In order to identify those genes we have computed an entropy-based numerical criterion (see Materials & Methods). Entropy is high if many experiments contribute equally to the overall score, it is low if a single (or few) experiment accounts for a large fraction of the score. For example, the gene *Serpina1b *has the top score (7.9, rank 1/15,277) in our study. This is due to a very high fold-change in a single experiment; consequently, entropy is low (1.17, rank 4,590/15,277). In contrast, other genes show more consistent alterations across many different studies, for example *Pdk4 *(6.7, rank 3/15,277) indicated by higher entropy (3.0, 167/15,277). Differential expression of *Pdk4*, a major regulator of glucose metabolism, has been found in fat, pancreatic islets and skeletal muscle but not in liver.

The thirty genes with highest scores are listed in Table 2 [The entire candidate list is given in Additional file 2].

**Table 2:** Top thirty T2DM candidate genes (out of 213).

**SourceName**	**mgi_symbol**	**entrezgene**	**refseq_dna**	**HUGO**	**Score**	**Entropy**
ENSMUSG00000071178	Serpina1a ; Serpina1b	20701	NM_009244	SERPINA1	7.899	1.167
ENSMUSG00000028639	Ybx1	22608	XR_003217 ; XR_003023 ; XR_001819 ; NM_011732		7.065	2.484
ENSMUSG00000019577	Pdk4	27273	NM_013743	PDK4	6.668	2.993
ENSMUSG00000005054	Cstb	13014	NM_007793	CSTB	6.11	2.746
ENSMUSG00000022878	Adipoq	11450	NM_009605	ADIPOQ	6.082	3.043
ENSMUSG00000031980	Agt	11606	NM_007428	AGT	5.912	2.052
ENSMUSG00000068220	Lgals1	16852	NM_008495	LGALS1	5.895	1.85
ENSMUSG00000026249	Serpine2	20720	NM_009255	SERPINE2	5.894	2.49
ENSMUSG00000031762	Mt2	17750	NM_008630	MT1X	5.67	2.72
ENSMUSG00000036427	Gpi1	14751 ; 676974	NM_008155		5.598	1.619
ENSMUSG00000037071	Scd1	20249	NM_009127	SCD	5.553	2.358
ENSMUSG00000025453	Nnt	18115	NM_008710	NNT	5.539	2.307
ENSMUSG00000016194	Hsd11b1	15483	NM_008288	HSD11B1	5.452	2.489
ENSMUSG00000026628	Atf3	11910	NM_007498	ATF3	5.446	2.676
ENSMUSG00000023087	Ccrn4l	12457	NM_009834	CCRN4L	5.434	2.333
ENSMUSG00000021190	Lgmn	19141	NM_011175	LGMN	5.225	2.588
ENSMUSG00000061780	Cfd	11537	NM_013459	CFD	5.158	2.429
ENSMUSG00000029657	Hsp110	15505	NM_013559	HSPH1	5.14	2.438
ENSMUSG00000025006	Sorbs1	20411	NM_178362 ; NM_001034964 ; NM_001034963 ; NM_001034962 ; NM_009166	SORBS1	5.092	2.639
ENSMUSG00000029309	Sparcl1	13602	NM_010097	SPARCL1	5.024	2.013
ENSMUSG00000024981	Acsl5	433256	NM_027976	ACSL5	4.912	2.868
ENSMUSG00000032018	Sc5d	235293	NM_172769	SC5DL	4.879	1.622
ENSMUSG00000035385	Ccl2	20296	NM_011333		4.856	1.764
ENSMUSG00000041417	Pik3r1	18708	NM_001024955 ; NM_011085	PIK3R1	4.852	3.008
ENSMUSG00000026003	Acadl	11363	NM_007381	ACADL	4.839	2.911
ENSMUSG00000006818	Sod2	20656	NM_013671	SOD2	4.746	3.127
ENSMUSG00000020027	Socs2	216233	NM_007706	SOCS2	4.682	2.57
ENSMUSG00000026687	Aldh9a1	56752	NM_019993	ALDH9A1	4.666	2.467
ENSMUSG00000020593	Lpin1	14245	NM_172950 ; NM_015763	LPIN1	4.639	2.285
ENSMUSG00000027690	Slc2a2	20526	NM_031197	SLC2A2	4.617	2.461

Adiponectin (*Adipoq*) has a score of 6.1 (rank 5/15,277) and the high entropy indicates a consistent behavior across data sets. *Adipoq *is a hormone from adipocytes that modulates insulin sensitivity and thus regulates glucose and lipid metabolism and energy homeostasis. Expression of *Adipoq *is reduced in obesity, certain genotypes are associated with increased risk of T2DM in humans [38]. The protein is secreted from fat tissue and has insulin-sensitising and anti-inflammatory properties. Additionally, we find strong changes in the expression in muscle both in human and mice. *Adipoq *is an oxidative regulator. The systemic oxidative stress causes the metabolism to share the burden from fat to muscle [39]. *Adipoq *is also responsible for the crosstalk between the three KEGG pathways '*PPAR signaling', 'Adipocytokine signaling' *and '*type II diabetes mellitus'*. It has been tested for transcriptional regulation but no binding to the TFs under study could be detected. A negative regulation has been described for *Tnf *that has not been tested in the underlying studies.

*Pdk4 *phosphorylates and inhibits pyruvate dehydrogenase complex thereby contributing to the regulation of glucose metabolism. Expression of this gene is regulated by glucocorticoids, retinoic acid and insulin. This is in accordance with a consistent differential expression in fat, muscle and pancreatic islets resulting in high entropy. On the other hand, possible regulation of the gene by *Hnf4a *and *Usf1 *is reported in liver.

Hydroxysteroid (11-beta) dehydrogenase 1 (*Hsd11b1*, score 5.5, rank 13/15,277) is a critical enzyme for cortisol metabolism. *Hsd11b1 *is increased in obese subjects and transgenic mice over-expressing *Hsd11b1 *develop visceral obesity [40]. Inhibition of *Hsd11b1 *decreases blood glucose in hyperglycaemic mice. Selective antagonists are currently developed and tested as anti-obesity and anti-diabetes drugs.

*Scd1 *(5.6, 11/15,277) is the rate-limiting enzyme in monounsaturated fatty acid synthesis. It has been shown to exert a critical role in hepatic lipogenesis and lipid oxidation. *Scd1 *knock-out mice are lean due to increased energy expenditure, show increased insulin sensitivity and are resistant to diet-induced obesity and liver steatosis.

Nicotinamide nucleotide transhydrogenase (*Nnt*, 5.5, 12/15,277) is a mitochondrial enzyme involved in proton transport into the mitochondrial matrix. *Nnt *was identified as a novel candidate gene in a quantitative trait locus for glucose intolerance [41]. *Nnt *has been recently shown to regulate insulin secretion in pancreatic beta cells. *Nnt *deficiency results in defective insulin secretion and inappropriate glucose homeostasis [42]. It has been proposed that *Nnt *detoxifies reactive oxygen species [43] implicating a possible role of *Nnt *in regulating ATP production in mitochondria and function of the ATP sensitive K+ channel Kir6.2 (*Kcnj11*) in insulin producing beta cells.

For eighteen genes only limited functional information is available as a basis for assessing a possible relationship to T2DM: *Ccrn4l, Serpina12, Htatip2, Mest, Pgcp, Tmsb4x, Angptl4, Mrpl33, Ndfip1, Yipf5, Tmem30a, Asnsd1, Oact5, Larp5, Thrsp*, *1810015C04Rik, 2310003F16Rik*, and *2610002J02Rik*. High genetic variation is known for *Pgcp *in mouse. *Serpina12*, a target of *Hnf4a*, is massively changed in liver and *1810015C04Rik *in pancreatic islets. Using the entropy criterion we observe medium to high entropy in these genes, like in *Ndfip1 *(entropy 2.9), what points to the fact that high scoring of these genes was not due to single outlier experiments but that these genes are truly affected by the disease and, thus, exhibit a high potential for further functional experiments.

### T2DM-GeneMiner web tool

In order to allow users to screen the disease potential of any given gene of interest we developed T2DM-GeneMiner, a web interface summarizing the results of our work (Figure 1, [35]). The user interface is shown for the well-known *Adipoq *and the resulting bar plots for two other genes, *Pdk4 *and *Cfd*, with lower content of available information. The resource is searchable by gene or protein IDs (for example Ensembl ID or gene symbol). The score distribution is shown as a bar plot and, where available, functional information is displayed. The two rightmost bars show the entropy, indicating uniform or specific score distribution, and the score. The red line at the score bar indicates the cut-off.

### Overlap to previous predictions of T2DM genes

**Figure 2:**
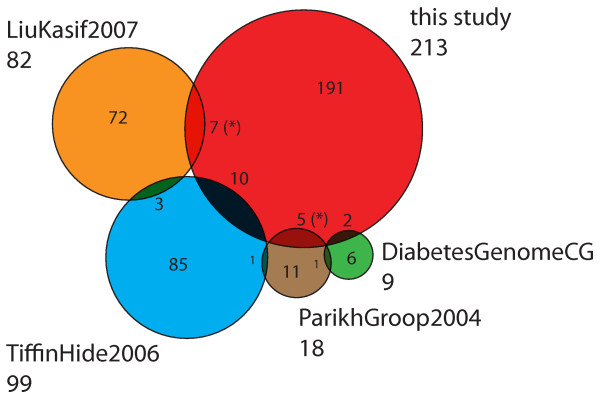
**Venn diagram of candidate gene lists.** Abbreviations relate to the following references: TiffinHide2006 [22], DiabetesGenomeCG [23], ParikhGroop2004 [47] and LiuKasif2007 [24]. One gene in TiffinHide2006 has been neglected for the count since no transcriptional information was available for that gene. (*) Two genes are counted twice because the intersection of LiuKasif2007 and our study shares those genes with ParikhGroop2004.

From fourteen genes in the OMIM description of T2DM (Diabetes mellitus, noninsulin dependent, #125853, [44]) five genes have a significant score in our study: *Retn*, *Gpd2*, *Vegfa*, *Irs2 *and *Tcf2 *(see Table 3). *Retn *represents an adipocytokine which has been implied to play roles in obesity, diabetes, and insulin resistance [45,46]. Interestingly, *Retn *is only deregulated in one of two studies involving adipose tissue. In contrast, differential expression for *Irs2*, *Vegfa *and *Tcf2 *was observed in pancreatic islets whereas *Gpd2 *did not show tissue-specific expression. Several previous studies have already published T2DM candidate lists allowing us to assess common content. The overlap to the list of the Diabetes Genome Anatomy Project [23], being also the source of some of the transcriptome data sets used for this meta-analysis [12-14], results in a P-value of 9.9E-03. Using the same resource, with a less conservative selection of data sets, Liu et al. identified 82 genes related to insulin signaling with an overlap of seven genes to our candidate list containing several strongly connected proteins (see below) [24]. More selective is a review of sequencing candidates leading to a P-value of 5.28E-13 [47]. In Tiffin et al. 99 candidates were published as partial overlap of several electronic candidate prediction methods [22]. This results in a P-value of 1.9E-05 comparing it with our list (Figure 2 shows a Venn diagram of the absolute gene numbers). In summary, the T2DM candidate gene list includes a small amount of candidate genes from previous studies and, further, leads to an additional set of 191 genes not identified in the other studies. Subtracting those genes for which we have disease information from the incorporated reviews our approach identifies 128 novel T2DM candidate genes.

**Table 3:** Results for T2DM OMIM genes.

**SourceName**	**Mgi symbol**	**Stumvoll Goldstein 2005**	**Dean McEntyre 2004**	**OMIM**	**PubMed Gene RIF**	**KO mice Jax**	**Nandi Accili 2004**	**score**	**entropy**	**significant gene**	**rank (out of 15,277)**
ENSMUSG00000012705	Retn			*	*			4.597	2.106	*	31
ENSMUSG00000026827	Gpd2			*				4.452	2.88	*	39
ENSMUSG00000023951	Vegfa			*	*			4.273	2.724	*	52
ENSMUSG00000038894	Irs2	*		*	*	*		3.907	2.112	*	82
ENSMUSG00000020679	Tcf2		*	*	*			3.175	1.605	*	176
ENSMUSG00000041798	Gck	*	*	*	*	*		3	1.585		234
ENSMUSG00000029644	Ipf1	*	*	*	*	*		3	1.585		234
ENSMUSG00000029556	Tcf1		*	*	*	*		3	1.585		234
ENSMUSG00000040136	Abcc8	*	*	*	*			2.795	1.848		325
ENSMUSG00000034701	Neurod5; Neurod1		*	*	*			2.393	1.48		608
ENSMUSG00000017950	Hnf4a	*	*	*	*			2.36	1.614		642
ENSMUSG00000024985	Tcf7l2			*	*			2.192	1.371		811
ENSMUSG00000037370	Enpp1			*	*			2.106	1.237		918
ENSMUSG00000027223	Mapk8ip1			*				1	0		3013

### Relation to monogenic mouse models for T2DM

A variety of genetic studies have been performed in the last decades. At least nineteen genetically engineered mouse models with T2DM phenotype have been studied in detail [2,3]. Of those, five genes show a significant score in our meta-analysis: *Slc2a4*, *Irs2*, *Ptpn1*, *Slc2a2 *and *Irs1*. Consistent with previous reports, the insulin-regulated glucose transporter GLUT4 (*Slc2a4*) is down-regulated in the insulin resistant state in adipose tissue but not in skeletal muscle. Likewise, down-regulation of *Irs2 *and the glucose transporter GLUT2 (*Slc2a2*) in pancreatic islets confirms previous reports and reflects deterioration of beta cell function in the course of insulin resistance and diabetes.

On the other side *Slc2a2 *is also changed in liver. *Ptpn1 *is expressed in all tissues showing only small fold-changes. Several genes from OMIM or KO-mice do not change at all on the expression level. This indicates that only the complete loss of the associated protein alters the system whereas the gene's expression is not altered in T2DM. For KO-mice we also see a strong tendency to genes only expressed in mice.

### Relation to human and rodent association and linkage studies

Recently, a total of nine candidate genes for T2DM have been identified and replicated in humans through multiple genome-wide association studies of common variants by using high-density SNP mapping approaches: *Cdkal *(score 0),*Cdkn2a *(score 0)*/Cdkn2b (*1.925, 1165/15277), *Fto *(1.798, 1364/15277), *Hhex *(1.213, 2456/15277), *Igf2bp2 *(0.855, 3555/15277), *Kcnj11 *(2, 1056/15277), *Pparg *(2.528, 500/15277), *Slc30a8 *(0.076, 8056/15277), and *Tcf7l2 *(2.192, 811/15277) [5-11]. Interestingly, none of these genes shows a high score in our meta-analysis, although *Pparg *and *Tcf7l2 *are significant on the less restrictive 0.01 level. On the other hand, from the data we could infer that *Fto *and *Hhex *act in pancreatic islets indicated by the T2DM-GeneMiner result for these genes. *Cdkal1 *and *Cdkn2a *are not expressed in the transcriptional studies. These genes show very low expression levels or might be active in tissues not included in our study. Since our meta-analysis approach takes into account several data sets from DNA microarrays, our candidate genes have a bias towards transcripts whose expression is changed in the context of T2DM. Moreover, the gene variants from association studies may not result in altered gene expression and, for most SNPs found in association studies, there is a lack of functional information since the variation mostly occurs in non-coding regions of the genes. In order to correlate the T2DM genes with genetic variation we plotted the number of known SNPs for the genes [see Figure 2 in Additional file 1]. No general tendency to highly variable genes is observable. Two genes of the candidate list show high variation, *Pgcp *(9,098 SNPs) and *Sorbs1 *(4,130). Particularly interesting is *Pgcp*, because it has not been related to T2DM before and its functional role is also undetermined.

**Figure 3:**
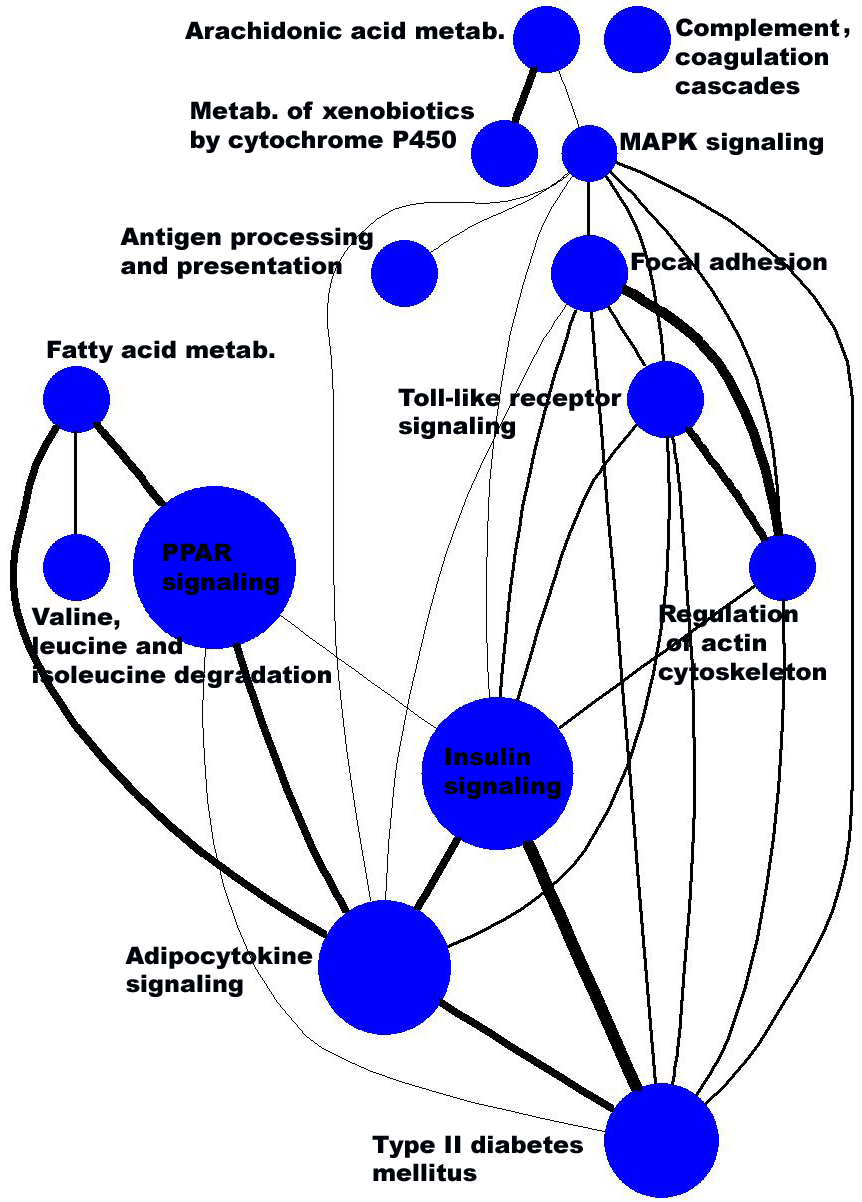
**Pathway crosstalk with respect to the T2DM candidate gene set.** Pathways were derived from the KEGG database. Each pathway has been weighted according to the total disease score reflected by the size of the nodes. Only pathways with a total score > 20 were selected for display. The thickness of the edges between the different pathway nodes reflects the overlap score derived from the sum of the scores of the overlapping genes. The graph was generated with the graphviz package [62].

A further issue of our study was the chromosomal localization of the T2DM genes. The marker genes are scattered over the entire mouse genome [see Figure 3 in Additional file 1]. Using the hypergeometric distribution on local sliding windows across the chromosome we could identify significantly enriched chromosomal regions. However, none of these regions convinced since they are sparsely occupied. For example on chromosome 2 with 2,249 genes and 15 T2DM candidates, a window of 20 genes containing two T2DM candidates leads to a P-value of 0.007. None of the windows of 20 genes contained more than three candidates. Rather conversely, we observe that T2DM affects a wide range of physiological phenomena spanning loci in the entire genome.

### Assessing functional annotation with enrichment analyses

Enrichment analyses based on the hypergeometric distribution were carried out in order to assess whether our T2DM candidate list is over-represented with respect to a certain functional category (Table 4). Categories on the physiological level comprise three major pathway resources (KEGG, Reactome, BioCyc) [28-30] and the GO tree [27]. Altogether, we have analyzed 4,555 gene sets, whereof 314 (6.9%) are significant with a P-value below 0.05 [see Additional file 3].

**Table 4:** Overview about the network level, e.g. gene set, resources used in the meta-analysis approach.

**Resource**	**Species**	**Resource content**	**Version**	**No. gene sets**	**Reference**
KEGG	mouse	pathway	09.01.2007	182	[29]
Reactome	human	pathway	19	691	[28]
BioCyc	human	pathway	9.1	169	[30]
OdomYoung2004	human	study of selected TF in liver and pancreas	publication	6	[18]
OdomYoung2006	human	study of selected TF in liver	publication	6	[17]
TransFac	mouse	sequence motifs for TF	10.2	187	[31]
GO molecular function	mouse	ontology	Ensembl 41	987	[56,57]
GO cellular component	mouse	ontology	Ensembl 41	350	[56,57]
GO biological process	mouse	ontology	Ensembl 41	1977	[56,57]

As greater parts of the metabolism are affected by T2DM, multiple pathways have a significant enrichment P-value. For example, in KEGG 45 out of 182 pathways have a P-value lower than 0.05. Table 5 shows the results of pathways with a P-value lower than 1.0E-04. Results for different pathways are not independent. For example, the 136 genes annotated with '*Insulin signaling pathway' *and the 46 genes annotated with '*type II diabetes mellitus' *share 32 genes. The first four pathways help to validate our significant gene set. '*PPAR signaling', 'Adipocytokine signaling' *and *'Insulin signaling pathway' *are well related to T2DM.

**Table 5:** Gene set enrichment of the most significant KEGG pathways.

**Pathway ID**	**SigSet**	**Set**	**Sig**	**All**	**P-value**	**Q-value**	**Pathway description**
path:mmu03320	13	69	213	15274	1.02E-11	1.37E-09	PPAR signaling pathway
path:mmu04920	12	73	213	15274	3.46E-10	1.66E-08	Adipocytokine signaling pathway
path:mmu04930	10	44	213	15274	3.69E-10	1.66E-08	Type II diabetes mellitus
path:mmu04910	13	128	213	15274	2.70E-08	9.09E-07	Insulin signaling pathway
path:mmu04612	6	38	213	15274	1.30E-05	0.000351	Antigen processing and presentation
path:mmu00280	6	44	213	15274	3.11E-05	0.000697	Valine, leucine and isoleucine deg.
path:mmu04610	7	67	213	15274	3.98E-05	0.000764	Complement and coagulation casc.

**All **are the genes under consideration, **Sig **the number of candidate genes, **Set **is the number of genes in the pathway under study and **SigSet **the overlap of genes in the pathway and the candidate genes. **P-values **were computed with the upper tail of the hypergeometric distribution indicating the probability of observing this overlap by chance. **Q-values **are the multiple testing corrected P-values [60,61].

Since we used several pathway resources in parallel, we can compare the findings for consistency, assuming the resources are independent. For example, we found enrichment of the KEGG pathway '*Fatty acid metabolism*' what is complemented by the BioCyc pathways '*fatty acid elongation – saturated*', '*fatty acid elongation – unsaturated*' and by the GO categories '*positive regulation of fatty acid biosynthesis*', '*positive regulation of fatty acid metabolism*', '*fatty acid binding*' and '*fatty acid oxidation*'. The KEGG pathway '*Complement and coagulation cascades*' is complemented by the Reactome pathways '*Initial triggering of complement*', '*Complement cascade*' and the GO categories '*defense response*' and '*complement activation, alternative pathway*'.

For 116 T2DM gene candidates there is information on the associated biochemical pathways according to the KEGG database. Whereas most genes (106) are associated with a single or a few (up to five) pathways, some genes exhibit a higher interconnection such as *Mapk1 *(22 pathways), *Pik3r1 *(19), *Aldh9a1 *(15), *Mapk9 *(11), *Sh3glb1 *(9), *Pla2q12a *(9), *Pkm2*, *Nfkbia*, *Dhrs7*, *Actb *(all 6). The importance of *Mapk1*, *Pik3r1*, *Rasa1 *and *Socs2 *is also supported by Liu et al. as members of an insulin signaling subnet derived from protein-protein-interactions [24].

In order to identify crosstalk between pathways we scored the pathways according to their counts and their overlap in the T2DM candidate list (Figure 3). An important module with a large number of genes represented in the T2DM list and a high overlap is visible with the pathways "*Insulin signaling*", "*Type II diabetes mellitus*", "*PPAR signaling*", "*Adipocytokine signaling*", and "*Fatty acid metabolism*" pointing to the interplay between obesity and insulin resistance. Another path of signaling action is activation of the RAS/RAF/MEK MAPK cascade resulting in cell growth and gene expression alterations expressed by the crosstalk between "*Type II diabetes mellitus*" and "*MAPK signaling*" pathways.

### T2DM-related protein-protein interactions

**Figure 4:**
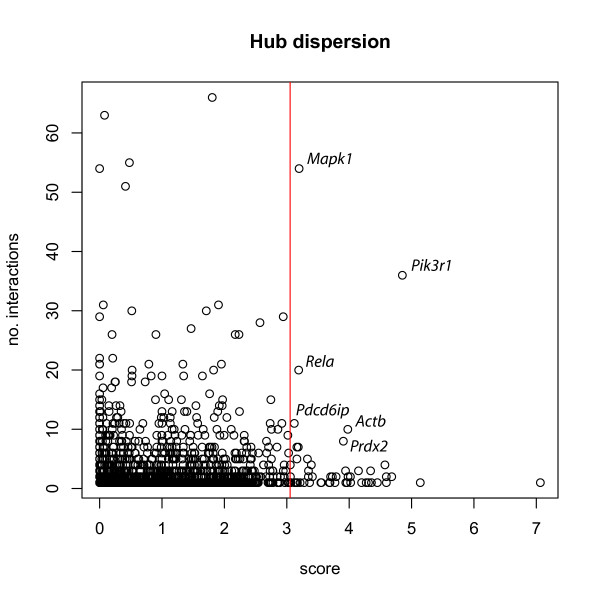
**Scatterplot of the number of mouse protein interactions in IntAct and the T2DM gene score. **The vertical red line indicates the significance cut-off value of the score. *Mapk1 *and *Pik3r1 *are highlighted as genes with more than 30 interactions.

Protein-protein interactions have been taken from the IntAct database denoting the number of interactions and interactors registered for the T2DM candidate genes. The ratio of interactors to interactions indicates whether the protein participates in big complexes or binds with single proteins. Figure 4 shows the number of interactions and the score for the genes under study. There is no trend for preferential selection of highly interacting genes in our T2DM candidate list. The high-scored genes comprehend a few genes with many interactions like *Mapk1*, *Pik3r1 *and *Rela *in mouse with more than 15 interactions. The large number of interactions of *Mapk1 *and *Pik3r1 *is consistent with their participation in many of the signaling pathways (Figure 3). *Actb*, *Cltb*, *Hspa5 *and *Grn *have more than 600 interactors, indicating big polymers. In human *Tsc22d1*, *Tnfrsf1b*, *Ndrq1 *and *Nme1 *have most interactions. *Lmna *is the only gene with more than 300 interactors.

Mapping the interactions on Ensembl genes and coerce the human net and mouse net we derive a graph with 5,179 nodes and 216,446 edges (data not shown). If we consider the edges between significant genes and their non-significant nearest neighbors we still have 1,471 nodes and 11,378 edges. This shows that the disease genes strongly interact with main physiological triggers and deregulate essential parts of the metabolic network. Reducing the interactions on the 213 T2DM genes we end up with 45 nodes and 167 edges [see Figure 4 in Additional file 1].

### T2DM-related gene regulatory network

In order to study the information content of the set of selected disease genes on gene regulation, we have analyzed a) the TFs present in our significant set and b) known target sets of TFs for enrichment. Analysis is often hampered because TFs are known to be expressed at a very low level and fold changes are commonly low. Moreover, many TFs are regulated by phosphorylation (e.g. *Foxa*'s) and/or ligand binding (e.g. *Ppar*'s). As a result, important core regulators including *Onecut1 *(score 1.2, rank 2461/15,277), *Hnf4a *(2.36, 642/15,277), *Tcf1 *(3, 243/15,277), and *Foxa2 *(1.4, 2055/15,277) are not in our candidate list.

Collecting TFs from Odom et al. [17,18], TransFac [31] and the GO category GO:0003700 in mouse and human with evidence codes IC, IMP, TAS or IDA we identify 490 TFs. Thereof 12 TFs received a high score in our T2DM set: *Srebf1*, *Tcf2*, *Rela*, *Ybx1*, *Cebpb*, *Nr1d2*, *Klf10*, *Nfil3*, *Ccrn4l*, *Atf3*, *Nme1 *and *Drap1*. *Srebf1 *and *Ybx1 *are expressed only in mouse but in every tissue. *Cebp*'s and *Srebp*'s are important regulators of lipid metabolism and adipogenesis and were found differentially expressed in the course of insulin resistance and T2DM. Consistent changes could be identified in the tissues under study (fat: all but *Nfil3*; liver: *Srebf1*, *Ccrn4l*, *Ybx1*, *Bhlhb2*, *Klf10*, *Nme1*; muscle: *Atf3*, *Klf10*, *Nme1*, *Nfil3*; pancreatic islets: *Ccrn4l*, *Atf3*, *Ybx1*, *Bhlhb2*, *Klf10*, *Nme1*, *Nfil3*). *Pparg *is expressed solely in fat where its expression is altered. In total, target sets of 187 TFs have been investigated as gene sets for enrichment analysis. Table 6 shows the TFs from Odom et al. [17] with significant P-value. For example, *Cebpa *is highly significant. It is expressed in adipose tissue and modulates the expression of leptin. *Cebpa *shows some correlation with the level of hyperglycemia in [16]. Alteration is also observable in liver.

**Table 6:** Gene set enrichment of significant TF target sets from Odom et al. [17].

**Transcription factor**	**SigSet**	**Set**	**Sig**	**All**	**P-value**	**Q-value**
ENSMUSG00000037025:FOXA2	33	738	213	15274	2.76E-09	0.0410
ENSMUSG00000017950:HNF4A	92	3812	213	15274	3.81E-09	0.0410
ENSMUSG00000029556:TCF1	29	846	213	15274	6.66E-06	0.0410
ENSMUSG00000043013:ONECUT1	30	1096	213	15274	0.000291	0.0410
ENSMUSG00000026641:USF1	25	1290	213	15274	0.0581	0.0410
ENSMUSG00000025958:CREB1	27	1794	213	15274	0.366	0.0694

The target sets with a P-value below 0.05 are displayed as an extract [see Additional file 3]. Column identifiers as in Table 4.

**Figure 5:**
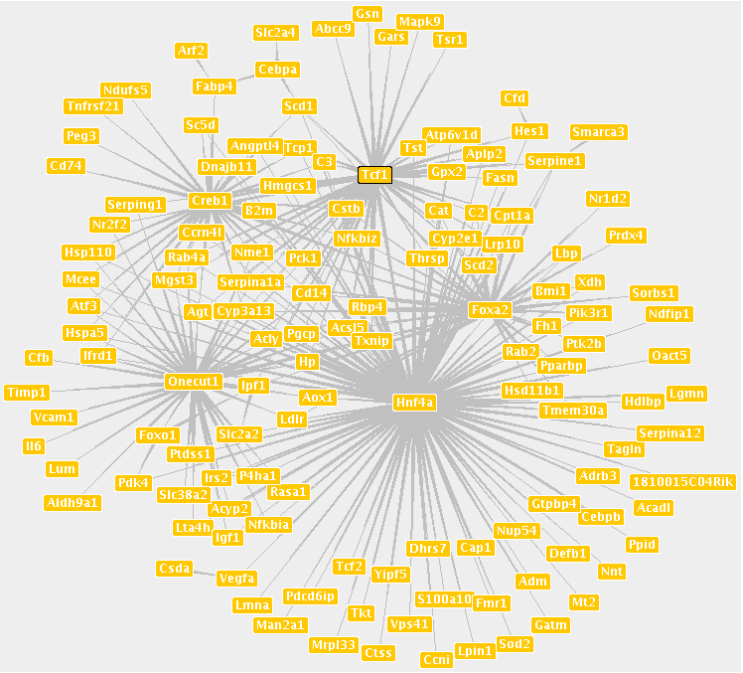
**Gene regulatory network composed of the significant genes.** Significant TFs and TFs with enriched target sets with respect to the T2DM candidate gene list. Thick ends of the arrows point to TFs, thin ends point to target genes.

A gene regulatory network comprising the regulatory interactions of the significant genes and the significant and enriched TFs is shown in Figure 5. Obvious are the five hubs, the core regulatory circuit derived from [17]. Well-regulated candidates can be identified like *Acly *and *Fabp4*. Target and regulator at the same time is *Ipf1*.

## Discussion

The first part of our study was devoted to the identification of genes related to T2DM using different heterogeneous data sources in different organisms. Genes have been scored in each individual study according to their disease relevance and an overall score across the different studies has been computed that reflects their total disease relevance. By this approach we were able to identify 213 genes that have a general disease relevance showing high scores in many different studies as well as genes that have a specific disease relevance expressing high scores in only a few studies.

In the second part of this work the computed T2DM gene set has been used to identify biological networks on different layers of cellular information such as signaling and metabolic pathways, a comprehensive gene regulatory network and protein-protein interactions.

Biological validity of the T2DM candidate set is assessed with a comparison to existing studies and disease gene repositories such as OMIM and genome wide association studies (GWA). The union of the medical reviews [1,48], genetic sources [2,3,44] and the PubMed hits [49] contains 481 genes with an overlap of 64 genes (30%) to our candidate genes. However, at present only a few genes have been identified through GWA in humans [5-11]. Since the contribution of most of the known risk alleles to the development of type 2 diabetes is rather small, one might conclude that many additional genetic factors are still unknown. Therefore, and since there is no unambiguous set of candidates that defines truly positive disease genes in a polygenic context, our analysis may provide guidance for future systematic investigation of candidate genes and further validation studies. Nevertheless, variants of *Pparg *and *Tcf7l2 *have been consistently found in recent GWA for T2DM and appear to be moderately significant in our meta-analysis as well.

It should furthermore be noted that GWA studies themselves show only little overlap. Two recently published meta-analysis studies on GWA [50,51] have an overlap of a single gene, PPARG, which is significant at the 0.01 level in our study.

Our scoring approach is very general. It combines genome-wide transcriptional studies from the Affymetrix GeneChip platform for obesity-induced T2DM, selective previous knowledge as well as gene lists derived from the biomedical literature.

In the study at hand each resource has the same weight in the computation of the score. The entire method, however, could be tuned towards a certain focus by introducing weights and computing weighted scores with weighted random background distributions, for example, if one is particularly interested in a certain tissue or in a certain cellular level of information (transcriptome, gene regulation, proteome). In order to weight the fold changes derived from transcriptome data we have used the information on the reproducibility of the signals (coefficient of variation from replicate experiments) and the information whether the gene is expressed in the study samples or not. We have found that this weighting procedure improves results in comparison to simple fold changes because, for example, genes that are not expressed in the study samples might also show high – but senseless – fold changes adding additional noise to the procedure. Alternative weights could be introduced by taking into account the genetic variation as number of SNPs, or the degree in the protein-protein interaction network to separate major players from redundant genes following the hypothesis, that key factors have to be well-connected.

In our approach we include both, genes with low but consistent expression changes across the different studies as well as strongly differentially expressed genes with respect to a single study. We used entropy as an indicator for measuring generality and specificity of a candidate gene with respect to the different studies. The correlation between the score and the entropy is 0.80 [a plot of the entropy versus the score is given in Figure 5 in Additional file 1]. However, most of our T2DM genes have high entropy and, thus, contribute to expression changes in many of the experiments.

Figure 2 reflects a limited overlap of the T2DM genes predicted by this study with those predicted by other bioinformatics methods. This difference can be explained by the differences in the data domain used for the predictions (for example, sequence data, gene expression data, PPIs) and differences in the methods themselves. The lack of overlap is not unique to this study and seems to be a common problem with any two prediction studies. In particular, one study – Tiffin et al. – compared seven different analysis methods and found that there was no gene common to all studies [22]. However, it should be noted that using the same data sets different bioinformatics methods are able two find consistent overlap (five and six out of seven studies) as was shown in Tiffin et al. [22]. We explored the pairwise overlap of different T2DM candidate approaches (including our study) [see Additional file 4]. The comparison exhibits two characteristics. Firstly, a common lack of commonality is observable resulting in the different data used and methods applied in the approaches. Secondly, our meta-analysis has a comparable performance with respect to biologically validated gene sets (highlighted rows: GWA and OMIM T2DM genes).

Despite these major sources of variation, there are further differences in analyzing microarray data with respect to chip platform and probe annotation. We have used the remapped probe annotation as provided by the R/Bioconductor software [37] which results in different sizes of the probe sets so that results can be different when using the annotation of the chip provider.

To assess the reliability of using mouse and human gene expression data we have compared the datasets from the two species separately. This analysis has to be stratified for the tissues under study. For human the data sets include skeletal muscle and pancreas. If we reduce mouse data to the same two tissues and perform the score evaluation on the two species separately we end up with 6,173 genes in total in the intersection and a correlation of 0.64 between the two score vectors. The human meta-analysis generates 91 significant genes and the mouse meta-analysis 31 genes. The overlap has 15 genes: *Abcc8, Adipoq, Gck, Irs1, Irs2, Irs4, Ptpn1, Slc2a2, Lpl, Pik3r1, Tcf1, Retn, Serpine1, Rbp4, B2m*.

A common approach in meta-analyses is to apply the same statistical test to congeneric studies and combine the resulting P-values by the Fisher method or Z-Score. We found this not practicable in our case, since the experimental sources are too heterogeneous. An extension to the web tool may open the way to introduce weights, chosen by the user.

**Figure 6:**
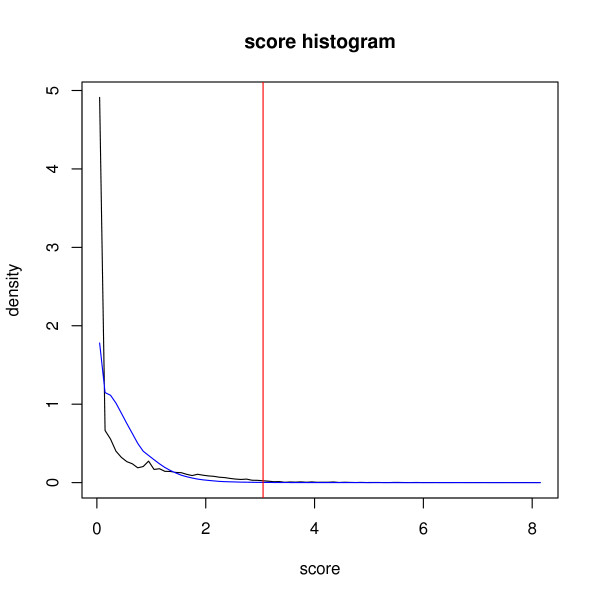
**Histogram of gene scores (black line) and background distribution of scores derived from Bootstrap**[**26**]**sampling (blue line).** The vertical red line marks the cut-off for the T2DM candidate gene list.

To contrast the different data sources a correlation heatmap is provided [see Figure 6 in Additional file 1]. In order to measure the dependency of the scoring method on published data – particularly review papers – we computed the correlation of the scores derived from the qualitative and quantitative data. The correlation is 0.07 indicating that the transcriptome data is rather independent of the published review knowledge. In the 'qualitative' category of our study, comprising reviews/OMIM, knock-out models and PubMedGeneRIF [1-3,19,44,47-49], we find 481 genes to be related with the disease. Only a small proportion (64 corresponding to 30%) of those genes were also found in our T2DM candidate list, so that the computed scores do not replicate literature knowledge to a dominating extent. This does not mean that our results lead to completely different results. Using a leave-one-out cross-validation with these studies we measured the significance of the overlap of each of these studies with our candidate list. This overlap can be quantified with the P-value derived from the upper tail of the hypergeometric distribution [52]. For all of the 'qualitative' reference sets we computed highly significant P-values (for example StumvollGoldstein2005: 7.09E-21, DeanMcEntyre2004: 5.28E-19, OMIM: 6.9E-15, PubMedGeneRIF: 6.73E-16).

Many existing T2DM data sets have not been incorporated in this study. For example, all transcriptome studies that were conducted on other microarray platforms than Affymetrix have been discarded because of compatibility issues. With the recent progress in merging gene expression data this limitation is soon questionable. Furthermore the selected data sets have a certain bias towards obesity-induced T2DM what is somewhat justified since obesity is a major risk factor. However, our study can be easily extended with additional sources of T2DM-relevant information. On the genetic level the results of QTL studies or, in human, association studies would upgrade the genetic component of the meta-analysis. Likewise to the OMIM source a sequence change does not necessarily lead to detection by expression change or a different criteria used in the study. E.g. a misfolded protein is not identifiable in this approach.

Furthermore, valuable information would be results from proteomic or metabolomic studies, but unfortunately data is still very sparse. Although the meta-analysis approach detects disease and candidate genes, it fails for some very specific well-known candidates. If a gene is only active at a medium level in only one tissue it will hardly be in our list. For example, *Pparg *(score 2.53, rank 500/15,277) is mainly expressed in fat tissue. Our general approach with a restrictive cut-off favors genes with either a consistent or a very strong alteration. However, with a less restrictive cut-off at the 0.01-quantil of the score we retrieve most of the known genes, but would increase the number of false positives to a high extent.

A simple enrichment analysis based on the hypergeometric distribution has been applied in order to characterize the T2DM set on the network level including pathways, regulatory networks and protein-protein interactions. In general, we find a high consistency of the results of the enrichment analysis when screening different databases. For enrichment we used a hypergeometric test and multiple testing corrections based on Q-values to keep the results comparable between the different databases. Alternative approaches might be useful if focusing on specific annotation. Particularly, for the GO database this approach does not take into account the specific graph structure. Furthermore, there is no unique structure available in the pathway databases. Here, for example up- or downregulation of the involved genes and control of the fluxes are important features and could be used to stratify the enrichment.

Protein-protein interactions are still very sparse or derived from high-throughput experiments with low overlap and low reproducibility so that results have to be carefully cross-checked. For example, we find a protein complex arising from one experiment of Collins et al. [53] with vague relationship to T2DM in the network of the candidate genes.

The gene regulatory network associated with the T2DM candidate set is generic in the sense that all interactions are displayed regardless whether the genes are expressed in a specific tissue or not. This network can be tuned towards tissue specificity by taking into account tissue-specific gene expression and other characteristics. Using tissue expression data sets [54] we can assess the representation of the different tissues in our T2DM candidate list. A total of 197 genes from the list are included in the tissue expression panel [41], where 140 (71%) are expressed in fat, 96 (49%) in muscle and 90 (46%) in liver. An intersection of 31% is expressed in all three tissues (data not shown).

There are further limitations in analyzing gene regulatory networks. Information of TF binding sites – besides computationally predicted sites – is sparse and the knowledge on target sets of TFs is limited. In Table 6 the P-values for six target sets of regulators are listed that have been derived from ChIP on Chip data. The Chip on Chip data might also help characterizing the 128 unknown T2DM genes as being potential TF targets. The overlap between this uncharacterized subset and the TF target sets are: *Hnf4a *50 genes, *Foxa2 *13 genes, *Usf1 *19 genes, *Tcf1 *11 genes, *Creb1 *19 genes and *Onecut1 *11 genes. However, this technique is still error-prone and generates a lot of false positive targets due to the different steps in the experiment. Commonly, we end up with large targets sets containing thousands of genes [17,18]. Here, new methods of computational analysis that combine ChIP on Chip-predicted targets with sequence analysis of their promoter regions have to be developed.

## Conclusion

We have identified a core set of 213 T2DM candidate genes by a meta-analysis of existing data sources. We have explored the relation of these genes to disease relevant information and – using enrichment analysis – we have identified biological networks on different layers of cellular information such as signaling and metabolic pathways, gene regulatory networks and protein-protein interactions.

## Methods

### Selection and integration of T2DM resources

Data sets were selected from heterogeneous sources that target different levels of cellular information. For each gene and each source we computed a numerical value that expresses its likelihood for being T2DM relevant. Data categories are either binary or quantitative.

Binary data was introduced by incorporating medical reviews, phenotype information (for example from knock-out genes), results from proteome analysis [1-3,19,44,48,49] as well as published candidate gene lists from previous studies or models [22,23,47,55]. Binary information was assigned according to the fact whether the gene had been identified in the study or not.

Quantitative data was incorporated by evaluating data from differential gene expression and time series microarray studies [12-16]. In order to extract comparable information across the different studies we used data from the same technological platform (Affymetrix GeneChip studies). Furthermore, in order to conduct standardized data normalization (see below) only studies were taken into account that published and provided the raw data (CEL file level) [see Additional file 5].

Genes do not act as individual units, they collaborate in overlapping pathways, the deregulation of which is a hallmark for the disease under study. In order to integrate pathway information and to derive cellular network information on the selected genes, we added functional annotation from pathway databases such as KEGG, Reactome, BioCyc [28-30], GO [27], protein-protein interaction databases such as IntAct [56] and databases on transcription factors (TFs) such as TRANSFAC [31].

Genetic variation of a gene was described with the number of associated SNPs. The number of SNPs in the coding and surrounding region of the gene is noted for mouse and human [57]. A particular biomedical interest is on genes that can be used for drug development. This characteristic has been previously assigned to the gene's ability to provide binding sites for biochemical well-characterized (i.e. druggable) compounds [58,59]. The selected candidates were evaluated with respect to this information. All collected information on the identified 213 T2DM candidate genes and further description of the data sets is given [see Additional file 2].

### Mapping of gene IDs

A central pre-requisite of any meta-analysis approach is the consolidation of the different ID types, for example coming from different organisms and from different versions of chips. We have used the Ensembl database [57] as the backbone annotation for all studies. IDs are mapped on their mouse Ensembl gene ID (version 41). Mapping and merging of the information has been done within R and the BioConductor package collection [33,34]. To ease the access for researchers we have added the more informative MGI marker symbols and HUGO ID's together with ENTREZ gene numbers and RefSeq IDs. In total, information on 15,277 Ensembl annotated genes has been mapped.

### Transcriptome data pre-processing and normalization

Affymetrix gene chip annotations were adapted from latest genome annotations [37] in version 8. Affymetrix data has been normalized with GC RMA using the R/BioConductor software platform [36]. For transcriptome studies that are targeting differential expression three bits of information are stored – the fold-change indicating the alteration of the gene when comparing the diabetic state with the normal state, the standard error of the fold-change computed from the replicated experiments in that study and the expression P-value (presence-call) that indicates whether or not the gene is expressed in the target samples under study. In time series studies we store the correlation between phenotypic characteristics, for example blood glucose, and the gene expression levels with the coefficient of variation and the expression P-value.

### Scoring T2DM relevance of genes across studies

In order to score the different categories of information, i.e. binary counts and quantitative gene expression values, for each category we summarized the scores of the individual experiments. For binary information the counts were grouped in sub-categories, for example knock-out mice described in two reviews only get a single count.

For quantitative information, the score of the *i*th gene in the *j*th study, s_*ij*_, was computed as follows:

$$s_{ij}=\{\begin{array}{cc} \left|\log2\left(r_{ij}\right)\right|\left(1-\frac{e_{ij}}{r_{ij}}\right)\left(1-p_{ij}\right), & p_{ij}\leq 0.1\text{ and }e_{ij}/r_{ij}\leq 1\\ 0, & \text{else} \end{array}.$$

Here, *r*_*ij *_is the fold change, *p*_*ij *_is the average detection P-value and *e*_*ij *_is the standard error of the ratio derived from the experimental replicates of the study. Thus, the fold change is weighted with its reproducibility across the experimental replicates and with the likelihood of the gene being expressed in the study's target samples. A similar formula applies for correlation studies:

$$s_{ij}=\{\begin{array}{cc} \left|c_{ij}\right|\left(1-v_{ij}\right)\left(1-p_{ij}\right), & p_{ij}\leq 0.1\text{ and }v_{ij}\leq 1\\ 0, & \text{else} \end{array}.$$

Here, *c*_*ij *_is the correlation to a certain phenotypic parameter, *v*_*ij *_the coefficient of variation of the gene's signal across experimental replicas. The formula is applied on the data of Nadler et al. [16]. Mice from three different strains (B6, BTBR and F2 intercrosses) are separated in five classes with increasing hyperglycemia. The Kendall rank correlation between the classes and the gene expression was calculated.

The total score of the gene was computed as the sum across all individual study scores.

### Sampling for significance

In order to assess the significance of the overall gene scores we generated random gene scores. For this bootstrap [26] we draw a random score from each study. The sum of the drawn study scores determines the score for a virtual gene. The distributions of the original scores (black line) and the random scores (blue line) are shown in Figure 6. Using the random distribution as background sample we assigned those genes as "significant" that are above the 99.9 percentile of the background distribution.

### Accounting for experimental study bias

For each gene, entropy of the score distribution was computed in order to quantify the relative influence of a certain study (for example a particular tissue) on the overall score. Let *s*_*ij *_be the score of the *i*th gene in the *j*th study, then *E*_*i *_is a measure for the uniformity of the score distribution over the individual experiments:

$$E_{i}=-\sum_{j}\frac{s_{ij}}{\sum_{k}s_{ik}}\log_{2}\left(\frac{s_{ij}}{\sum_{k}s_{ik}}\right).$$

Entropy is low if a single study has a major contribution on the overall score. On the other hand, entropy is high if most of the studies account equally for the score. A plot of the entropy versus the score is given in Figure 5 in Additional file 1.

### Identification of disease related networks using enrichment analyses

Disease related networks were investigated with four different types of network information – biological pathways [28-30], protein-protein interaction networks [56], gene regulatory networks [17,18,31] and functional annotation using GO annotations [27] (see Table 4). These networks define – by annotation – groups of associated genes. The hypergeometric distribution compares the overlap between our superset and the gene group to the overlap of a random selection of two gene sets with the same size [52]. Thus we were able to assign each annotation item (pathway, GO term etc.) a P-value that reflects enriched occurrence of candidate genes. In case of GO terms we include only genes with evidence codes IC, IMP, TAS or IDA to rely on the same confidence level as in the above mentioned resources. We correct P-values for multiple testing using Q-values following Storey for the control of the false discovery rate [60,61].

The same method we use in the leave-one-out cross-validation. Our qualitative studies are the benchmark for our scoring approach. The scoring, including a notional candidate set, is calculated without the respective qualitative study. The hypergeometric distribution of the qualitative study gene set and the notional candidate set assigns a P-value. This P-value reflects the success of the score to identify the genes from the qualitative study.

## Abbreviations

T2DM: type-2 diabetes mellitus; GO: gene ontology; TF: transcription factor; ID: identifier; SNP: single nucleotide polymorphism; GWA: genome-wide association.

## Authors' contributions

AR collected and processed the data, implemented and conducted the meta-analysis and wrote the manuscript. HA-H contributed with biological interpretation of the results and with writing the manuscript. RH designed and led the study, performed data analysis and contributed to the manuscript.

## Supplementary Material

Additional file 1 Additional figures. Supplementary figures for the manuscript. Click here for file

Additional file 2 Candidate genes. A tabular version of the web tool data for the T2DM candidate gene list of 213 genes. Click here for file

Additional file 3 Significant categories. The significant categories of the enrichment analysis. Click here for file

Additional file 4 Overlap of different candidate gene sets. A table showing the overlap of different candidate gene sets for T2DM. Click here for file

Additional file 5 Source description. Describing the sources of information used in the web tool and [Additional file 1]. Click here for file
