# Supplementary material for: Meta-Analysis Approach identifies Candidate Genes and associated Molecular Networks for Type-2 Diabetes Mellitus
Source: BMC Genomics. 2008 Jun 30;9:310. doi: 10.1186/1471-2164-9-310 (PMC2515154; doi:10.1186/1471-2164-9-310)
Supplement: Additional file 1 — Additional figures. Supplementary figures for the manuscript. [file 1471-2164-9-310-S1.pdf]

Score converges to true positives

Supplementary Figure 1

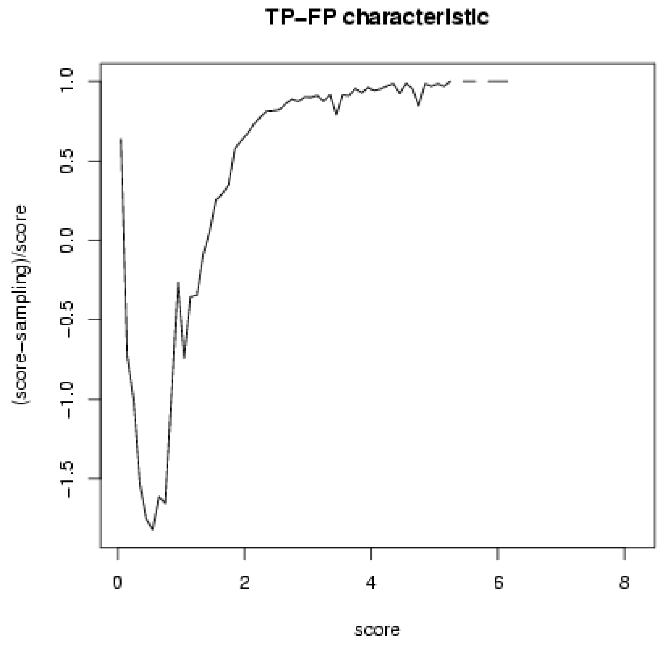

Compare the sampled false positive rate to the assumed true positives. The image is derived from figure 5 in the article. It shows on the y-axis the formula $y = \frac{x - s}{x}$ with x the gene score and s the sampled background distribution at x.

Genetic variation

Supplementary Figure 2

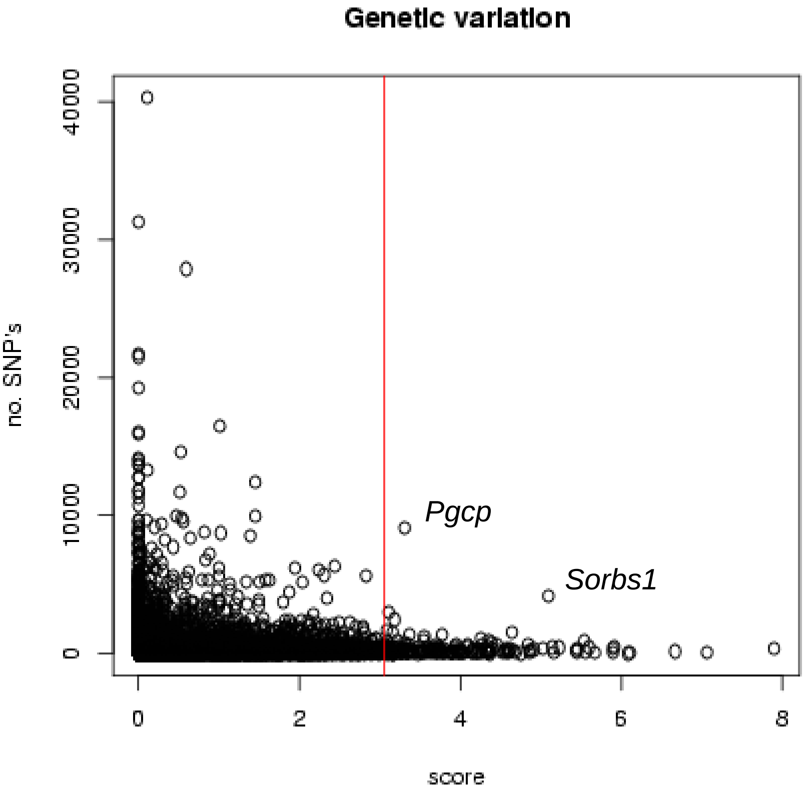

On the y-axis is the number of single nucleotide polymorphisms in the coding and surrounding region of the gene. A general tendency of disease genes to high genetic variation is not observable. This survey only relates the number of varied nucleotides. No conclusion is possible about single polymorphisms. The red line indicates the cut off for the candidate genes.

Chromosomal localisation of T2DM genes

Supplementary Figure 3

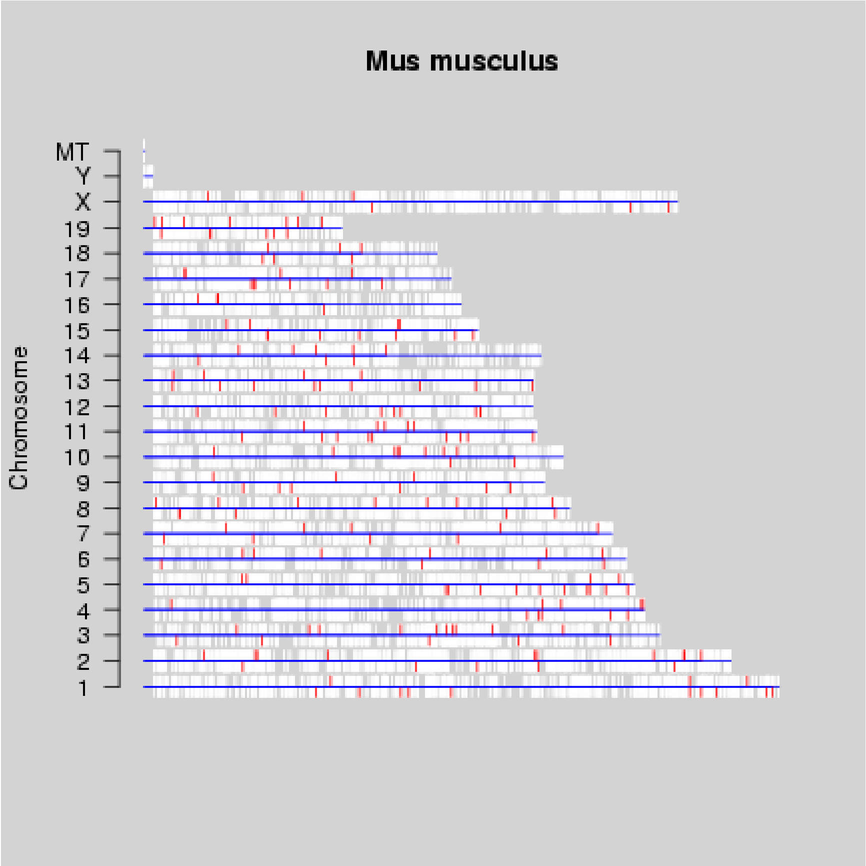

Chromosomal location of the significant genes. In a picture of the geneplotter package in R/BioC the set of significant genes has been marked with red. The significant genes are spread over the whole genome. It indicates no genomic region can be accountable for the heritable prevalence.
To look for new interesting regions, we used a sliding window approach. Using a window size of 20 genes we let the window slide over a chromosome and at every position the overlap between the 20 genes and the set of significant genes is calculated.

Protein-protein-interaction

Supplementary Figure 4

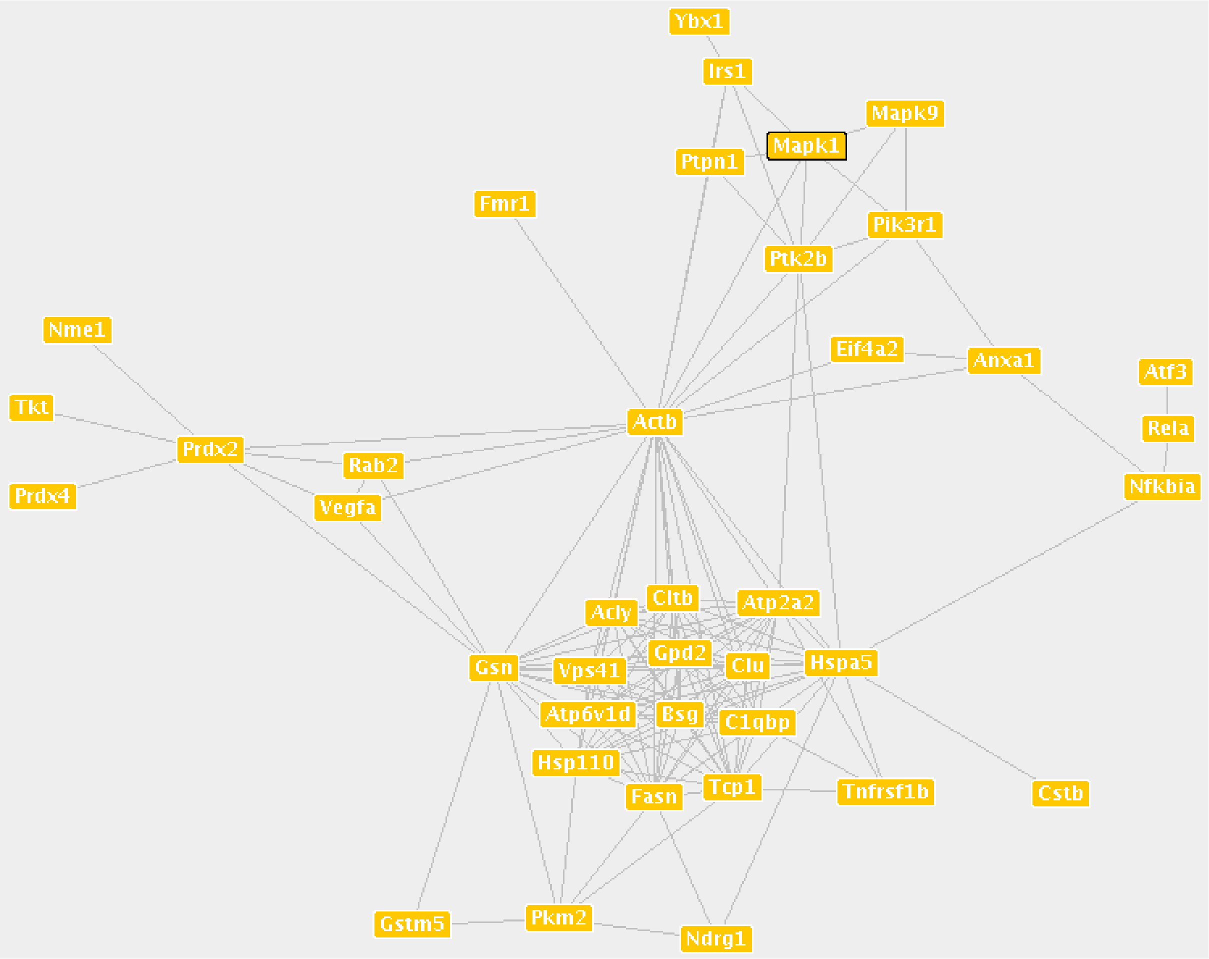

Protein interaction network of the significant gene list. The arcs are the interactions in IntAct between significant genes. The interaction network in mouse and human have been united. In the upper part we find Mapk1 as an intermediator between Pik3r1 and Irs1. In the lower part is a cluster stemming from a polymer binding in mouse synapses.

Dispersion of entropy

Supplementary Figure 5

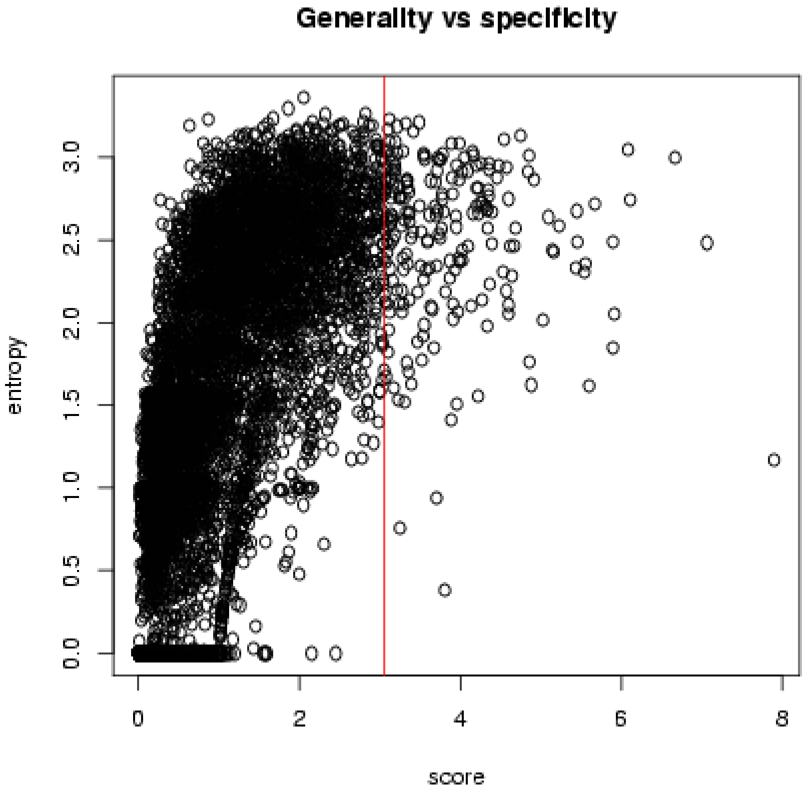

In the upper right corner are our target genes. Related to T2DM or consistent and strong alteration in expression. In the lower left corner are genes with strong alteration specific e.g. to tissue or species. The red line indicates the cut off for the candidate genes.

Correlation of data sources

Supplementary Figure 6

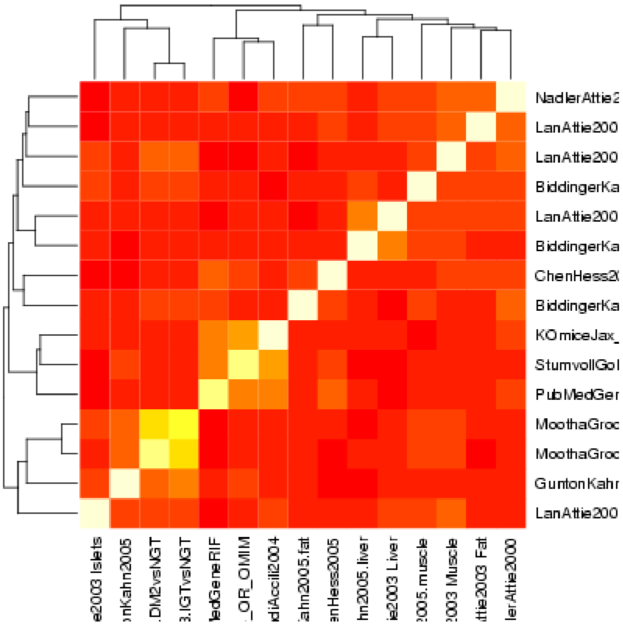

Heatmap of the scorepoint correlations between the sources. Both categories of sources are intermingled.
