# Supplementary material for: Meta-Analysis Approach identifies Candidate Genes and associated Molecular Networks for Type-2 Diabetes Mellitus
Source: BMC Genomics. 2008 Jun 30;9:310. doi: 10.1186/1471-2164-9-310 (PMC2515154; doi:10.1186/1471-2164-9-310)
Supplement: Additional file 4 — Overlap of different candidate gene sets. A table showing the overlap of different candidate gene sets for T2DM. [file 1471-2164-9-310-S4.pdf]

Supplementary Table 3 Pairwise overlap of different T2DM candidate approaches.

	study	study(lower)	Z2008	F2007	OMIM	TH2006	DGCG	PG2004	KM2004	LK2007
**this_study**	***	213	0	1	5	9	2	5	12	7
**this_study (lower cut-off)**	213	***	1	5	12	20	4	13	12	25
**Zeggini2008**	0	1	***	1	0	0	0	1	1	0
**Frayling2007**	1	5	1	***	3	0	0	2	2	0
**OMIM**	5	12	0	3	***	1	0	6	6	2
**TiffinHide2006**	9	20	0	0	1	***	0	1	4	3
**DiabetesGenomeCG**	2	4	0	0	0	0	***	1	1	0
**ParikhGroop2004**	5	13	1	2	6	1	1	***	7	2
**Kitano2004**	12	38	1	2	6	4	1	7	***	20
**LiuKasif2007**	7	25	0	0	2	3	0	2	20	***

This_study: 0.001 significance level; this_study (lower cut-off): 0.01 significance level; Zeggini2008, Frayling2007: two meta-analyses of GWA studies; OMIM: T2DM genes according to OMIM; TiffinHide2006, DiabetesGenomeCG, ParikhGroop2004, LiuKasif2007: candidate studies using different data sets; Kitano2004: genes used for a physiological model of T2DM.
