# Supplementary material for: Meta-Analysis Approach identifies Candidate Genes and associated Molecular Networks for Type-2 Diabetes Mellitus
Source: BMC Genomics. 2008 Jun 30;9:310. doi: 10.1186/1471-2164-9-310 (PMC2515154; doi:10.1186/1471-2164-9-310)
Supplement: Additional file 5 — Source description. Describing the sources of information used in the web tool and [Additional file 1]. [file 1471-2164-9-310-S5.pdf]

Descriptions of the columns in the Meta-Study

All the mapping of the gene identifiers and processing has been done to Ensembl Release 41 (Oct 2006). T2DM stands for type 2 diabetes mellitus.

Annotation: different gene identifiers

To give the biomedical researcher fast and convenient access to their favourite genes, four different types of identifiers are annotated to the main identifier.

ensembl

The main ID and backbone of the study: mouse ensembl gene ID. In Excel/OpenOffice scalc you can click on the ID and get the dedicated Ensembl webpage displayed in your browser.

mgi_symbol

The mouse MGI markersymbol from Jackson Labs corresponding to the mouse ensembl gene ID.

entrezgene

All the mouse NCBI Entrez ID's corresponding to the mouse ensembl gene ID.

refseq_dna

All the corresponding mouse RefSeq ID's corresponding to the mouse ensembl gene ID.

HUGO

The HUGO identifiers of the human homologs to the mouse ensembl gene ID.

Qualitative information

To use current medical knowledge, we analysed reviews from the literature addressing the disease. To amend the reviews we give the OMIM genes and genes for the respective knock-out mice with a T2DM phenotype. Most of the following sources provide a simple list of identifier.
A "*" is set to indicate a hint for the gene involvement in the disease. The cell is empty in all other cases.

StumvollGoldstein2005

Medical point of view on T2DM. In the list the homologs in the mouse are used.
(Stumvoll, Goldstein et al. 2005)

DeanMcEntyre2004

Medical (NCBI) point of view on T2DM: The book "The genetic landscape of diabetes" available on the NCBI homepage. In the list the homologs in the mouse are used.
(Dean and McEntyre 2004)

OMIM

The genes, that are linked to T2DM by the OMIM project. In the list the homologs in the mouse are used. MIM Number: #125853: 12.06.2006
(OMIM 2000)

PubMedGeneRIF

In the NCBI GeneRIF of the respective genes certain keywords like "type 2 diabetes" appear.
(Mitchell, Aronson et al. 2003)

KOmiceJAX

The genes for the respective knock-out mice and transgenic mice from Jackson Labs which are linked to the phenotype of NIDDM/T2DM.
(Jackson Labs 2005)

NandiAccili2004

The genes for the respective mouse models which are linked to the phenotype of T2DM in the review.
(Nandi, Kitamura et al. 2004)

ChenHess2005

The peptides secreted by white adipose tissue in rat. They are potential signalling proteins and thus relevant for the disease.
(Chen 2005)

DiabetesGenomeCG

The Diabetes Genome Anatomy Project denotes candidate genes on its homepage.
(mult. 2002)

ParikhGroop2004

The authors discuss several candidate genes.
(Parikh and Groop 2004)

TiffinHide2006

The intersection of seven electronic candidate prediction methods.
(Tiffin, Adie et al. 2006)

KitanoMuramatsu2004

Member genes of a physiological disease model.
(Hopkins and Groom 2002; Kitano, Oda et al. 2004)

High-throughput gene expression data

Since several years microarrays are used for genome-wide screening. In this sense several attempts have been made to identify the genes relevant for T2DM. We incorporate five of these datasets for mouse and human and up to four tissues. The transcriptional information is measured with Affymetrix GeneChips and listed in three columns for every source.

MoothaGroop2003

A differential expression analysis for gene expression in skeletal muscle of swedish males (human). For the two subtypes the same control group of 17 persons has been used. In IGTvsNGT the gene expression of eight persons with impaired glucose tolerance is compared to 17 persons with normal glucose tolerance. In DM2vsNGT the gene expression of 18 persons with T2DM is compared to 17 persons with normal glucose tolerance.
Three columns are given: fold-change between diabetic and control classes, standard error of the fold-change and P-value of the presence tag. The presence tag is calculated according to Affymetrix methods.
(Mootha, Lindgren et al. 2003)

GuntonKahn2005

Gene expression in pancreatic islets of caucasians (human). Five diabetic subjects are compared to seven control persons.
Three columns are given: fold-change between diabetic and control classes, standard error of the fold-change and P-value of the presence tag. The presence tag is calculated according to Affymetrix methods.
(Gunton, Kulkarni et al. 2005)

LanAttie2003

Gene expression of obese mice with (BTBR) and without (B6) T2DM is compared in four tissues (fat, liver, muscle, pancreatic islets). Two technical replicates are hybridized. The mice were 14 weeks old. Three columns are given: fold-change between diabetic and control classes, standard error of the fold-change and P-value of the presence tag. The presence tag is calculated according to Affymetrix methods.
(Lan, Rabaglia et al. 2003)

BiddingerKahn2005

The gene expression of obese and diabetic mice is compared with lean and not-diabetic mice from the 129 strain.
Three columns are given: fold-change between diabetic and control classes, standard error of the fold-change and P-value of the presence tag. The presence tag is calculated according to Affymetrix methods.
(Biddinger, Almind et al. 2005)

NadlerAttie2000

Mice from three different strains (B6, F2, BTBR) are separated in five classes with increasing hyperglycemia. The Kendall rank correlation between the classes and the gene expression in fat is calculated. There are no replicates in this study. For the single replicate three to four mice are used. The mice were 14 weeks old.
Three columns are given: correlation between classes and gene expression, coefficient of variation and P-value of the presence tag. The presence tag is calculated according to Affymetrix methods.
(Nadler, Stoehr et al. 2000)

Gene regulation information

OdomYoung2004

Targets of the transcription factor HNF4α. ChIP on Chip study in human pancreatic islets and liver. In the study the P-value of the binding affinity is given. The reference used a cut-off of 0.05. The transcription factor HNF4α is assumed to be involved in the disease. So all his targets are of interest. The human targets have been mapped on mouse homologs.
(Odom, Zizlsperger et al. 2004)

OdomYoung2006

The same authors published a work about six transcription factors in liver. For each gene the regulators are listed. The human targets have been mapped on mouse homologs.
(Odom, Dowell et al. 2006)

TransFac

For the genes the regulators in mouse from the TransFac database are listed; version 10.2.
(Matys, Kel-Margoulis et al. 2006)

Functional information

Pathways listed in databases provide network and kinetic modeling information on a quantitative/qualitative level. The annotated information is often only readable in the computer. The pathway ID’s are to long for the cells in Excel/OpenOffice scalc.

KEGG

For KEGG mouse pathways the number of annotated pathways and the list of pathway ID's is given. Date of the database: 09.01.2007.
(Kanehisa and Goto 2000)

Reactome

For Reactome human pathways the number of pathways and the pathway ID is given. The human genes have been mapped on mouse homologs. Version of the database: 19.
(Joshi-Tope, Gillespie et al. 2005)

BioCyc

For BioCyc human pathways the number of pathways and the pathway ID is given. The human genes have been mapped on mouse homologs. Version of the database: 9.1.
(Romero, Wagg et al. 2004)

GO

For GO categories the number of categories and the GO ID is given.
(Ashburner, Ball et al. 2000)

Genetic variation

SNP

The number of SNP's in the region of the gene are given according to Ensembl for mouse and human.

Protein interaction

IntAct

Protein-protein-interaction is annexed with IntAct in the version of 09.03.2007. For every gene the number of interactions and interactors in IntAct is listed for mouse and human. The difference hints for compounds or polymers.
(Hermjakob, Montecchi-Palazzi et al. 2004)

DruggableGenome2002

An electronic prediction of genes appropriate as medical agent.
(Hopkins and Groom 2002)

DruggableGenome2005

An electronic prediction of genes appropriate as medical agent.
(Russ and Lampel 2005)

Gene evaluation

Score

The score calculated for every gene by the T2DM specific sources.

Entropy

The entropy for the score. Indicates whether the score is derived from only one (statistical outlier?) or more sources.

References

References

Ashburner, M., C. A. Ball, et al. (2000). "Gene Ontology: tool for the unification of biology." Nature Genetics **25**: 25-29.

Biddinger, S. B., K. Almind, et al. (2005). "Effects of diet and genetic background on sterol regulatory element-binding protein-1c, stearoyl-CoA desaturase 1, and the development of the metabolic syndrome." Diabetes **54**(5): 1314-23.

Chen, X. a. C., S.W. and Pannell, L.K. and Hess, S. (2005). "Quantitative Proteomic Analysis of the Secretory Proteins from Rat Adipose Cells Using a 2D Liquid Chromatography-MS/MS Approach." J. Proteome Res. **4**(2): 570-577.

Dean, L. and J. McEntyre (2004). The Genetic Landscape of Diabetes, NCBI.
Gunton, J. E., R. N. Kulkarni, et al. (2005). "Loss of ARNT/HIF1beta mediates altered gene expression and pancreatic-islet dysfunction in human type 2 diabetes." Cell **122**(3): 337-49.
Hermjakob, H., L. Montecchi-Palazzi, et al. (2004). "IntAct: an open source molecular interaction database." Nucleic Acids Res **32**(Database issue): D452-5.
Hopkins, A. L. and C. R. Groom (2002). "The druggable genome." Nat Rev Drug Discov **1**(9): 727-30.
Jackson Labs. (2005). "Human Disease and Mouse Model Detail for NIDDM."
Joshi-Tope, G., M. Gillespie, et al. (2005). "Reactome: a knowledgebase of biological pathways." Nucl. Acids Res. **33**(suppl 1): 428-432.
Kanehisa, M. and S. Goto (2000). "KEGG: Kyoto Encyclopedia of Genes and Genomes." Nucleic Acids Research **28**(1): 27-30.
Kitano, H., K. Oda, et al. (2004). "Metabolic syndrome and robustness tradeoffs." Diabetes **53 Suppl 3**: S6-S15.
Lan, H., M. E. Rabaglia, et al. (2003). "Gene Expression Profiles of Nondiabetic and Diabetic Obese Mice Suggest a Role of Hepatic Lipogenic Capacity in Diabetes Susceptibility." Diabetes **52**(3): 688-700.
Matys, V., O. V. Kel-Margoulis, et al. (2006). "TRANSFAC and its module TRANSCompel: transcriptional gene regulation in eukaryotes." Nucleic Acids Res **34**(Database issue): D108-10.
Mitchell, J. A., A. R. Aronson, et al. (2003). "Gene indexing: characterization and analysis of NLM's GeneRIFs." AMIA Annu Symp Proc: 460-4.
Mootha, V. K., C. M. Lindgren, et al. (2003). "PGC-1alpha-responsive genes involved in oxidative phosphorylation are coordinately downregulated in human diabetes." Nat Genet **34**(3): 267-73.
mult. (2002). Diabetes Genome Anatomy Project.
Nadler, S. T., J. P. Stoehr, et al. (2000). "The expression of adipogenic genes is decreased in obesity and diabetes mellitus." PNAS **97**(21): 11371-11376.
Nandi, A., Y. Kitamura, et al. (2004). "Mouse models of insulin resistance." Physiol Rev **84**(2): 623-47.
Odom, D. T., R. D. Dowell, et al. (2006). "Core transcriptional regulatory circuitry in human hepatocytes." Mol Syst Biol **2**: 2006 0017.
Odom, D. T., N. Zizlsperger, et al. (2004). "Control of pancreas and liver gene expression by HNF transcription factors." Science **303**(5662): 1378-81.
OMIM. (2000, 04.10.2005). "Online Mendelian Inheritance in Man, OMIM (TM)." from http://www.ncbi.nlm.nih.gov/omim/

Parikh, H. and L. Groop (2004). "Candidate genes for type 2 diabetes." Rev Endocr Metab Disord **5**(2): 151-76.
Romero, P., J. Wagg, et al. (2004). "Computational prediction of human metabolic pathways from the complete human genome." Genome Biology **6**(1:R2): 17.
Russ, A. P. and S. Lampel (2005). "The druggable genome: an update." Drug Discov Today **10**(23-24): 1607-10.
Stumvoll, M., B. J. Goldstein, et al. (2005). "Type 2 diabetes: principles of pathogenesis and therapy." The Lancet **365**: 1333-1346.
Tiffin, N., E. Adie, et al. (2006). "Computational disease gene identification: a concert of methods prioritizes type 2 diabetes and obesity candidate genes." Nucleic Acids Res **34**(10): 3067-81.
